# Ocean climate and seal condition

**DOI:** 10.1186/1741-7007-3-9

**Published:** 2005-03-28

**Authors:** Burney J Le Boeuf, Daniel E Crocker

**Affiliations:** 1 Institute of Marine Sciences and Department of Biology, University of California, Santa Cruz, California, 95064, USA; 2 Department of Biology, Sonoma State University, Rohnert Park, California, 94982, USA

## Abstract

**Background:**

The condition of many marine mammals varies with fluctuations in productivity and food supply in the ocean basin where they forage. Prey is impacted by physical environmental variables such as cyclic warming trends. The weaning weight of northern elephant seal pups, *Mirounga angustirostris*, being closely linked to maternal condition, indirectly reflects prey availability and foraging success of pregnant females in deep waters of the northeastern Pacific. The aim of this study was to examine the effect of ocean climate on foraging success in this deep-diving marine mammal over the course of three decades, using cohort weaning weight as the principal metric of successful resource accrual.

**Results:**

The mean annual weaning weight of pups declined from 1975 to the late 1990s, a period characterized by a large-scale, basin-wide warm decadal regime that included multiple strong or long-duration El Niños; and increased with a return to a cool decadal regime from about 1999 to 2004. Increased foraging effort and decreased mass gain of adult females, indicative of reduced foraging success and nutritional stress, were associated with high ocean temperatures.

**Conclusion:**

Despite ranging widely and foraging deeply in cold waters beyond coastal thermoclines in the northeastern Pacific, elephant seals are impacted significantly by ocean thermal dynamics. Ocean warming redistributes prey decreasing foraging success of females, which in turn leads to lower weaning mass of pups. Annual fluctuations in weaning mass, in turn, reflect the foraging success of females during the year prior to giving birth and signals changes in ocean temperature cycles.

## Background

Multidecadal fluctuations, or biological regime shifts, have basin-wide effects on sea surface temperature (SST) and upwelling that are associated with large-scale changes in biological productivity [1,2]. They are similar to the 3–4 year cycles of the El Niño Southern Oscillation (ENSO) but on longer time scales, and as background states, may amplify ENSO effects [3]. The Pacific Decadal Oscillation (PDO), which addresses the North Pacific/North American sector, comprises events that persist for 20 to 30 years [4,5]. In the 20^th ^century, "cool" PDOs prevailed from 1947 to 1976 while "warm" PDOs were recorded from 1925 to 1946 and from 1977 through at least the mid-1990s. Chavez and colleagues [6], focusing on the last two regimes, report that in the mid-1970s the Pacific changed from a cool "anchovy regime" (ocean temperatures colder than normal, shallow thermocline and strong upwelling, increased nutrient supply and zooplankton and overall productivity, high catches of anchovies and salmon, low sardine abundance) to a warm "sardine regime" (ocean temperatures warmer than normal, deep thermocline and weak upwelling, decline in zooplankton and salmon and overall low productivity, high sardine abundance, low anchovy catch), which lasted until the middle to late 1990s before shifting back to an anchovy regime. The changing ocean temperatures associated with these regimes exert strong effects on marine food webs [7]. In the northeastern Pacific many marine birds and mammals flourish during anchovy regimes and are nutritionally stressed during sardine regimes. It is well documented that short-cycle ENSOs cause drastic reductions in survival and reproduction of several marine mammal species in the temperate waters of the Pacific [8]. Non-migrating sea lions and fur seals are especially vulnerable to ocean warming because nursing females alternate feeding and nursing, which ties them closely to their coastal rookeries and results in pup starvation when prey move out of the area [9]. It is not known, however, to what degree these processes impact deep-diving marine mammals that obtain their prey far below the surface as they range widely in the open ocean of the northeastern Pacific far from the coast.

The condition of newly weaned northern elephant seals, *Mirounga angustirostris*, is expected to be sensitive to these processes because of the location where adults feed and the close link in energy exchange between mother and pup. In this species weaning weight reflects total energy transfer from the mother because pups feed solely on mother's milk prior to weaning. Because the mother gives birth annually to a single pup and fasts during lactation, her pup's weight at weaning correlates positively with her resource accruing ability at sea during the eight-month period of pregnancy [10,11]. The mean weaning weight of the colony reflects the general foraging success of parturient females in that year. It follows that mean weaning weight may oscillate annually with ocean processes that exert strong effects on marine food webs and impact prey availability. A similar logic has been used in studies of the southern congener, *M. leonina*, that breeds and feeds in Antarctic waters [12-16]

**Figure 1:**
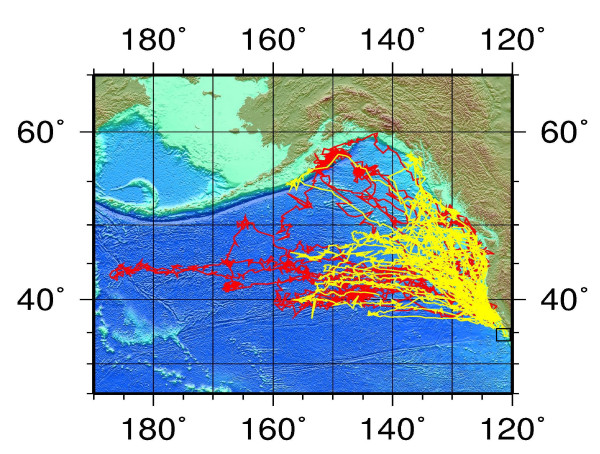
**Foraging location of adult females. **Rectangular mercator projection of spring (yellow) and fall (red) migration tracks of 35 satellite-tagged female elephant seals from Año Nuevo, California (black lined square), during the period 1995–1999. Adapted from previous studies [17,26].

Northern elephant seals are among the deepest diving marine mammals. Females disperse widely in the northeastern Pacific twice a year, from 38 to 60°N and from the coast to 188°E (Fig 1), foraging at modal depths of 300–600 m and maximum depths of 1500 m [17] on prey such as mesopelagic squid and Pacific hake, *Merluccius productus *[18]. After continuous foraging over the 8-month gestation period (mid-April to mid-December), females return to rookeries along the coasts of Mexico and California to give birth and nurse their pups. In four weeks a pup triples its birth weight and is weaned when the mother returns to sea in February for a 2 1/2 month post-partum bout of foraging to recover the one third of the body mass lost during nursing and begin the next pregnancy [10,19].

Milk energy transferred to the pup varies with its sex and the age, mass, blubber reserves and overall condition of the mother [11]. By maternal condition, we mean the total body energy available to the female at the time of parturition. On average, 48.0 ± 3.0% (3490 ± 490 MJ) of a female's body reserves is depleted during lactation. The size and blubber reserves obtained by the female during her biannual foraging migrations determine the level of reproductive expenditure in the subsequent breeding cycle. Pup weight at birth and weaning is correlated positively with the mother's age and mass [19]. Male pups are about 8% heavier than females at birth and at weaning [20], suggesting that males require more maternal energy than female pups. Pregnant females are capital breeders that store energy in blubber laid down over months of continuous foraging at sea and expend much of this energy later in reproduction [21]. Acquisition of insufficient body reserves owing to low foraging success directly impacts reproductive expenditure resulting in low weaning weight of pups [11]. Additional data supporting these points are reported in studies of southern elephant seals [13,22].

Here we show that annual weaning weights of northern elephant seal pups and resource accrual of adult females associated with a rookery in central California fluctuate with ocean temperature cycles.

## Results

### Weaning mass

**Figure 2:**
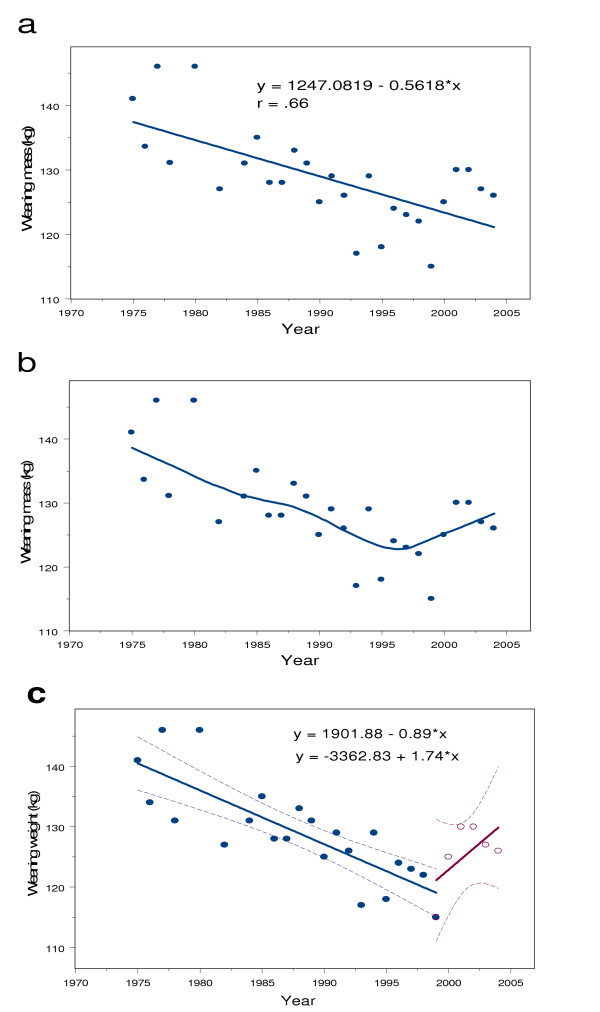
**Fluctuations in annual weaning mass of pups. **a) Least squares regression shows a significant decline in weaning mass over the entire study period. b) A Loess plot smoothes the data and reveals two functions, a declining slope and an increasing slope with the breakpoint between the two occurring in the late 1990s. c) A Least squares regression line reveals a significant decline in weaning mass from 1975 to 1999 (solid circles); the regression line from 1999 to 2004 (open circles) shows increasing weaning mass values. The dotted lines are the 95% confidence limits in kilograms.

The pattern of fluctuation in weaning weight over the years was similar in both sexes even though males were, on average, 5% heavier than females (overall mean of 131.7 ± 24.8 kg for males and 125.4 ± 21.4 kg for females). The regression of weaning mass on year showed a statistically significant decline in both males (F = 9.59, df = 1, 25, P = 0.0048) and females (F = 7.50, df = 1, 25, P = 0.0112). The regression slopes of males and females were not significantly different from each other (t = 0.11, df = 50, P > 0.05). The weaning mass data of both sexes, combined, which we address in the rest of this paper, declined significantly from 1975 to 2004 as illustrated by the scatter plot and regression of weaning mass against year in Fig. 2a (F = 19.7, df = 1, 25, P = 0.0002).

A Loess smoothing plot, however, reveals two distinct slopes (Fig. 2b). Mean weaning weight declined significantly from 1975 to the mid to late 1990s, decreasing from highs of 141 kg in 1975 and 146 kg in 1977 and 1980, to lows of 117, 118 and 115 kg in 1993, 1995 and 1999, respectively. A linear regression line through the data over the 24 year period from 1975 to 1999 (Fig. 2c) shows a significant mean decline of 0.89 kg/yr (F = 38.5, df = 1, 20, P = 0.0001). A significant decline is also observed if the end data point is anywhere in the interval 1995–1998. Similarly, the decline is statistically significant when years with a sample size of less than 30 are omitted, i.e. 1975, 1976, 1977 and 1982 (F = 25.93, df = 1, 16, P = 0.0001). In the late mid 1990s, the declining trend ends abruptly and the mean weaning weight increases up to 126 to 130 kg in the new century (Fig. 2b). Although the slope of weaning weight from 1999 to 2004, shown in Fig. 2c, is positive, it is not statistically significant (F = 2.12, df = 1, 4, P > 0.05) owing in part to the small sample size. The slope of weaning mass versus year, however, varied significantly between the two regimes (ANCOVA, F = 5.21, df = 1, 19, P = 0.03).

**Figure 3:**
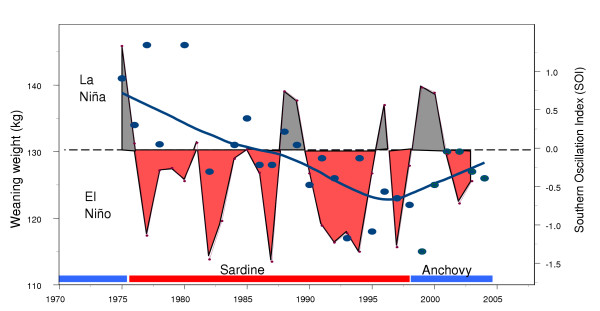
**Weaning mass in relation to ocean temperature cycles. **A Loess plot (blue line) of mean cohort weaning weight (closed circles) in relation to the sardine (thick red line) and anchovy (thick blue line) decadal regimes and the Southern Oscillation Index (SOI) showing colder than normal La Niñas (black) and warmer than normal El Niños (red).

The period of declining weaning weight was associated with higher than normal ocean temperatures associated with the long-term sardine decadal cycle and the prevalence of El Niños in the short-term ENSO cycles (Fig. 3). Weaning weight declined increasingly with the tenure of the sardine regime, a trend that ended abruptly with the shift to the anchovy regime in the late 1990s. With the regime shift, a reverse trend to increasing weaning weights is evident. Mean weaning weight between the two regimes was not significantly different (mean sardine period = 129 ± 8.2 kg vs mean anchovy period = 127.6 ± 2.3 kg) (t = 0.38, df = 23, P > 0.05) but this analysis is confounded by the declining slope of weaning mass over the course of the sardine regime and the small and incomplete sample size for the anchovy regime. A similar lack of association was found between weaning mass and PDO.

Declining weaning weight over the course of the decadal regime from 1975 to the late 1990s is consistent with the overlapping pattern of short-term ENSO cycles during this period, represented in Fig. 3 by annual mean monthly values of the standardized Southern Oscillation Index (SOI) [23]. This was clearly a period of extended warmth in the Pacific. Warm El Niños were more frequent than cold La Niñas, the El Niños of 1982–83 and 1997–98 were the most intense of the century, and the El Niño in the early 1990s (1991–1995) was unusually long. The global temperatures in 1997 and 1998 were the warmest years on record [24]. The lowest weaning weights were observed from 1993 to 1999, an unusually warm period dominated by two El Niños coming at the end of the sardine regime.

The decline in weaning mass was not linearly related to increasing ocean temperatures during the sardine regime. Although the period from 1975 to 1999 was warmer than usual, ocean temperatures did not increase significantly over the course of this period, as revealed by SOI and PDO annual averages regressed on year (F = 0.02, df = 1, 27, P > 0.05 and F = 0.08, df = 1, 28, P > 0.05, respectively). Consequently, a linear regression plot reveals that annual mean weaning mass was not closely associated with annual SOI (F = 0.13, df = 1, 24, P > 0.05) or annual PDO averages (F = 0.45, df = 1, 25, P > 0.05). It is notable, however, that the mean weaning weight over the period 1990–1999, which included the longest El Niño and the most intense one of the century, was 122.8 ± 4.8 kg, significantly lower than the mean weaning weight of 136.4 ± 7.2 kg recorded in the period 1975–1985, which included only the strong El Niño of 1982–1983 (t = 4.78, df = 16, P = 0.0002).

### Female foraging success: foraging duration and mass gain

To examine the relationship between weaning weight, ocean temperature and female foraging success, we used the spring post-breeding foraging trip duration, and mass gain over this period, as the metric of foraging success. During this foraging trip females can recover approximately 85% of the mass lost during parturition and nursing. We assume that foraging success in the spring is associated with and reflects foraging success in the fall. The duration of the fall foraging trip, however, is an unsuitable measure of foraging effort because its length is set by gestation, and is relatively invariant at 225.7 ± 4.3 days (N = 10).

**Figure 4:**
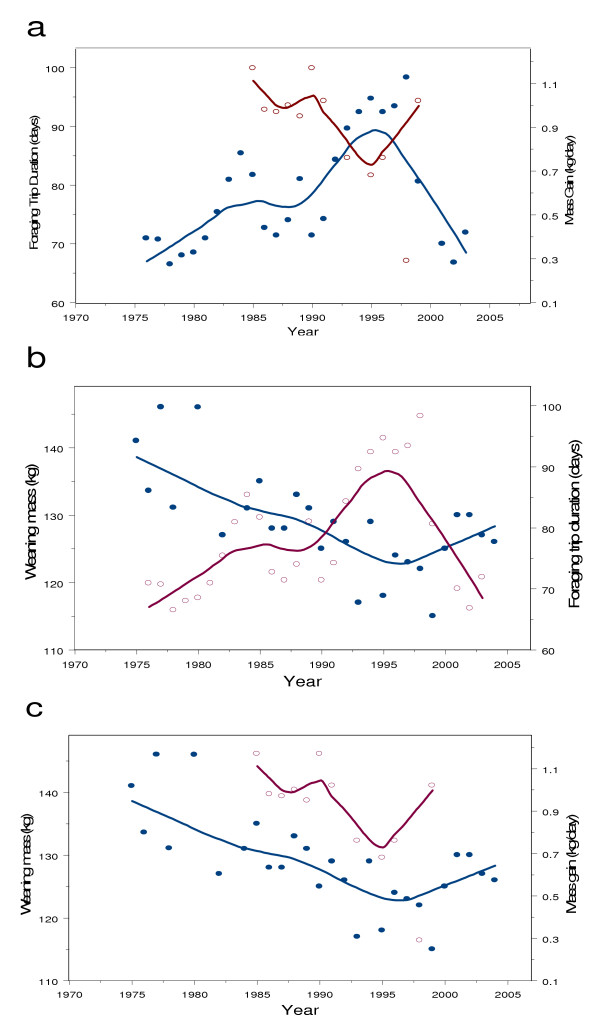
**Foraging effort and foraging success of adult females. **a) Loess plots of foraging trip duration of females (blue line, closed circles) show an inverse relationship with mass gain at sea (red line, open circles). b) A Loess plot shows that weaning mass (blue line, closed circles) is inversely related to foraging trip duration of females (red line, open circles). c) Weaning mass (blue line, closed circles) covaries with mass gain of adult females (red line, open circles).

The duration of spring foraging trips of females was inversely related to mass gained (Fig. 4a). The regression of mass gain on foraging duration was statistically significant (F = 22.3, df = 1, 10, P = 0.0008). Over the course of the sardine regime, post-partum females spent increasingly more time attempting to accrue resources [25,26], increasing the mean foraging trip duration by approximately 36% from the late 1970s to the mid to late 1990s (Figs. 3, 4a). With the shift to the anchovy regime in the late 1990s, foraging durations decreased from 90 days or more at sea to approximately 70 days at sea from 2001 to 2003, the same level observed at the beginning of the sardine regime. Association of foraging trip duration with shorter term El Niño cycles is also evident, such as the longest foraging trip and lowest mass gain occurring in 1998, an exceptionally strong El Niño year.

Mass gain during foraging trips was inversely related to trip duration (Fig. 4a), decreasing from approximately 1 kg/day at sea during the late 1980s to approximately 0.7 kg/day in 1993–1996, to a low of 0.29 ± .36 kg/day in 1998, and returning to 1 kg/day in 1999. In spite of small annual sample sizes, variation around the overall mean was small (mean = .90 ± 0.23 kg/day, n = 52).

Weaning weight of pups was inversely related to the foraging trip duration of females (Fig. 4b). Linear regression of pup weaning mass on female foraging duration shows that this relationship was statistically significant (F = 10.10, df = 1, 22, P = 0.0044). Weaning weight and mass gain of females covaried in time (Fig. 4c) but the sample size was small and the association was not statistically significant (F = 2.87, df = 1, 10, P > 0.05).

### Colony composition and sex ratio

The Año Nuevo colony increased over the course of the study period from 605 pups produced in 1975 to 2500 pups produced in 2004. The decline in weaning mass from 1975 to 1999 was not associated with changes in the age composition of the colony such that more young females gave birth to smaller pups. Rather, the opposite was observed; mean age at primiparity of females increased over the study period from a mean of 3.7 years in the early 1970s [27] to over 4 years in the 1990s.

The decline in weaning mass was not due to a sex ratio increasingly biased to smaller female pups. There were marginally more females in the sample from 1977 to 1987 (51% of 583 pups), when mean weaning weight was highest, and more males in the sample from 1990 to 1999 (51.6% of 1278 pups) when mean weaning weight was lowest. The sex ratio of weaned pups in the entire colony has not deviated significantly from unity during the last four decades [20].

## Discussion

Our data show that the weaning mass of northern elephant seal pups varies with ocean temperature cycles. Weaning mass declined over the course of an unusually warm 24-year period that coincided with the warm decadal or sardine regime and included several long and strong short-term warm ENSO events. The decline in weaning mass ended abruptly with the regime shift to the cold anchovy period. Weaning mass increased with the return to the cold regime but monitoring throughout an entire anchovy period is required to reveal the entire pattern and determine whether the positive trend of increasing mass continues.

The link between ocean temperature and weaning weight is strengthened by the finding that weaning mass was inversely related to the foraging effort of females and positively related to the mass they gained. Over the course of the warm period from 1975 to about 1999, females spent increasingly longer periods searching for food; the more time spent searching, the less mass they gained. Evidently, females attempted to compensate for reduced foraging success by staying at sea longer searching for food. This would buffer the effect of reduced foraging success and would obviously complicate any expectations of a linear relationship between SOI and weaning mass and rates of female mass gain and pup weaning mass. The ability of a given female to extend her pre-implantation foraging trip, however, is limited by her need to implant in time to give birth during the breeding season and is impacted by her weaning date the previous year. Females do not have the ability to compensate in this way during the gestational foraging trip. The relationship between SOI and weaning mass is further confounded by changes in colony age distribution. As the colony has aged, weaning mass should increase based on our understanding of the relationship between maternal mass and investment [11]. SOI helps define the strength and duration of ENSO cycles but it does not predict weaning mass of pups well except during unusually strong or long El Niños. We emphasize that it is important that we detected such a strong trend in pup weaning mass despite the trip duration compensation by females and the changing colony demographics. This implies that reduction in resources were greater than could be compensated for by increased foraging duration in any given year or by increasing maternal body size with age. By this logic, it is reasonable that we did not observe a linear relationship from SOI and PDO to rate of foraging success to pre-implantation trip duration to weaning mass.

It is clear that foraging effort was greatest and foraging success was lowest at the end of the sardine regime during the late 1990s. With the return to the cold regime in the late 1990s, female foraging effort returned to the level observed in the mid-1970s, the time of the previous regime change.

We reason that warmer than usual ocean temperatures influenced elephant seal prey, either through recruitment, competitive advantages or predation, effecting a redistribution or diminution of populations. As a result, female seals were less successful forging, and consequently, at parturition and during lactation, they had less energy to transfer to their young. This logic is supported by observations during El Niño years. During the strong El Niño of 1997–98, foraging females spent more time travelling from one prey patch to another and they spent less time on each prey patch than in non-El Niño years [26]; mass gain in 1998 was by far the lowest value we have recorded. Moreover, enduring effects of these "hard times" are suggested by the observation that female pups born in 1983, a strong El Niño year, postponed breeding for the first time relative to adjacent cohorts in the years 1977 to 1986 [25].

The link between seal condition and physical measurement of ocean temperature is complicated. The progressive decline in weaning mass over the course of the sardine regime is not simply explained by increasing ocean temperatures. The SOI and PDO indices did not increase systematically over the course of this period. Evidently, other factors such as the duration of exposure to warming or warm temperature spikes during ENSO cycles overlaid on a warm decadal cycle may have been operative. Anthropogenic perturbations, or global warming, may amplify the effect of these natural cycles [28]. In any case, the temporal link between temperature changes, effects on prey, and the weaning mass of pups is unclear. Substantial lag effects may operate, as has been observed in the link between the PDO index and salmon catches [4]. Moreover, female seals spend 10 of the 12 months of the year foraging and it is not clear whether foraging is affected during all or part of this time. An additional complexity is the difficulty of separating the effects of short-term ocean temperature cycles from the long-term cycles. For example, in addition to the long period of warm water associated with the sardine regime, an unusually long El Niño occurred during the early 1990s that had a more severe impact on marine mammals and birds than was predicted from its strength. Overall, the data suggest that the duration and persistence of warm water may have a cumulative negative effect on seal prey and indirectly on seal foraging behaviour and, ultimately, on pup weaning mass. It is also likely that the long-term cycle, as background state, may amplify or dampen the effect of short-term cycles, e.g. the impact of an El Niño on seal prey and foraging may be amplified during a sardine regime and dampened during an anchovy regime. Monitoring weaning mass and foraging behaviour over the entire course of the current cold anchovy regime may shed light on these issues.

This study shows that understanding the relationship between ocean climate and biological processes depends on having a long and complete time-series. In the present study, single year records yielded estimates of weaning mass ranging from 147 to 115 kg, a 28 % change from lowest to highest estimate. Even samples of five consecutive years would have provided quite disparate estimates, e.g. means of 140, 130 and 121 kg for the years 1975–1980, 1985–1990 and 1995–2000, respectively. This study, ranging over 29 years, not only provides a more valid and reliable estimate of weaning weight of northern elephant seals from central California, it also presents a wide range of observed values and the circumstances associated with this variability. Even so, the present study suggests that representative sampling of pup weaning weight should cover at least two successive multidecadal regimes, about 50 continuous years!

## Conclusion

Elephant seals, one of the deepest divers in the ocean and a migratory mammal that spends 83% of the year feeding far from the coast in pelagic waters beyond the thermocline in the modal temperature range 4.2–5.2°C [29], are impacted by ocean warming. Females spend more time searching for prey and are less successful in acquiring it when ocean temperatures are warmer than usual. Body stores laid down during long foraging periods at sea set limits on energy transfer to pups. Mean weaning mass of pups, consequently, fluctuates from year to year and reflects the impact of cyclic warming on the availability of elephant seal prey in the northeastern Pacific in that year. Weaning mass declined significantly during the unusually warm period from 1975 to 1999. It is well documented that offspring mass at the time of nutritional independence affects the probability of survival in many animals and is a crucial variable in population growth [22,30-32].

It is notable that the decline in elephant seal weaning mass we report here paralleled in time the 60 to 80% decline of Steller sea lions, *Eumetopias jubatus*, northern fur seals, *Callorhinus ursinus*, and non-migratory harbor seals, *Phoca vitulina*, in the northern Gulf of Alaska that began in the late 1970s and only abated to approximately 5% in the late 1990s [33-36]. Our results provide additional support for the hypothesis that nutritional stress [37] is a partial explanation for population declines such as these.

Lastly, our findings support the assertion that monitoring aspects of ecosystems, in addition to monitoring climate data, facilitates early identification of regime shifts [38].

## Methods

### Weanling measurements

We weighed 2750 pups within 10 days of weaning. Mean sample size of all years in the interval 1975–2004 was 108.6 ± 68.2 and the range was 5–224. Sample sizes were smallest in the years 1975 (6), 1976 (5), 1977 (14) and 1982 (5). In every other year the sample size was 30 or more. Weanlings were not weighed in 1979, 1981 or 1983.

Selection of individuals for weighing was unbiased and based primarily on convenience with one exception. Pups weighing less than 50 kg were excluded from the analysis because they were orphaned before weaning, suckled briefly and were only 10 kg above mean birthweight [20], were in poor physical condition and injured, and were judged unlikely to survive to four months of age [39]. They were not excluded because their mothers had insufficient resources to nurture them.

We weighed all pups on the peripheries of female harems to minimize disturbance to the rookery. We attempted to weigh weanlings as soon as they were accessible to the weighing team; pups usually exited harems within a day or two after weaning.

Each animal was captured and weighed in a nylon restraint bag lifted by a winch attached to a scale suspended from an aluminium tripod. Scale precision was ± 1 kg. Since weanlings fast and lose mass, weaning mass was estimated based on known rates of mass lost per day. We used the following equation for back-calculating mass at weaning:

*Mass at weaning = Measured mass·(e^k·d^)*,

where k = .00596 and d = number of days between weaning and weighing [19].

### Estimates of foraging success

Foraging trip duration of 410 adult females was measured annually from 1976 to 2003 (except for 2000) during the post-partum spring foraging trip. Mean sample size was 15.2 ± 12.2 per year (range = 3–54). We recorded departure time from the rookery and return to it by identifying individuals from a combination of dye and bleach marks, VHF transmitters and satellite tags. During the period 1985 to 1999, mass gain of 52 females (3 to 6 per year) was determined over the spring period at sea by weighing the females on the rookery before they departed and when they returned [21]. We determined the foraging trip duration of 10 adult females during the fall migration, which encompasses the entire gestation period.

### Colony demographics

Each year we determined the sex ratio of pups produced. We monitored changes in colony size and age composition with weekly censuses, identifying animals flipper-tagged at weaning [40].

### Ocean temperature

Thermal dynamics of the ocean were estimated from monthly Southern Oscillation Index (SOI) values [41] and from the Pacific Decadal Oscillation (PDO) Index [42,43].

### Statistical analysis

We used the nonparametric Loess plot in the Axum 7 software package (MathSoft Engineering and Education, Inc., Cambridge, Massachusetts) to identify slope changes and smooth the scatter plots of annual fluctuations in pup weaning mass, female foraging duration and female mass gain [44]. This method uses locally weighted linear regression to smooth data. The span, which determines the bandwidth as a fraction of the range of the x-axis values of the data, was 0.5. Local linear fits were used and the weight function was symmetric.

We present the actual P values for tests that were statistically significant. The alpha level (probability of Type I error) was set in advance at 0.05.

## Authors' contributions

BL conceived the study, established data collection protocols, analyzed data, drafted the manuscript, and revised the manuscript after peer review. DC participated in data collection, data analysis and the final revision. Both authors read and approved the final manuscript.
